# Supplementary material for: Electroconvective viscous fingering in a single polyelectrolyte fluid on a charge selective surface
Source: Nat Commun. 2023 Nov 17;14:7455. doi: 10.1038/s41467-023-43082-9 (PMC10656491; doi:10.1038/s41467-023-43082-9)
Supplement: Supplementary file 1 — Supplementary Information [file 41467_2023_43082_MOESM1_ESM.pdf]

Supplementary Information:

Electroconvective Viscous Fingering in a Single Polyelectrolyte Fluid on a Charge Selective Surface

by Jeonghwan Kim, Joonhyeon Kim, Minyoung Kim and Rhokyun Kwak

Contents:

Supplementary Note 1. Fluid properties of polyelectrolyte solutions 2

Supplementary Note 2. Electroconvective viscous fingering in various polyelectrolytes. 5

Supplementary Note 3. The derivation of the finger velocity 6

Supplementary Note 4. pH/PAA concentration profiling 7

Supplementary Note 5. The derivation of the Deborah number 8

Supplementary Figures

- **Supplementary Figure 1** 9
- **Supplementary Figure 2** 10
- **Supplementary Figure 3** 11
- **Supplementary Figure 4** 12
- **Supplementary Figure 5** 13
- **Supplementary Figure 6** 14
- **Supplementary Figure 7** 15
- **Supplementary Figure 8** 16
- **Supplementary Figure 9** 17
- **Supplementary Figure 10** 18
- **Supplementary Figure 11** 19
- **Supplementary Figure 12** 20
- **Supplementary Figure 13** 21

Supplementary Tables

- **Supplementary Table 1** 22
- **Supplementary Table 2** 23
- **Supplementary Table 3** 24
- **Supplementary Table 4, 5** 25

Reference 26

Supplementary Note 1. Fluid properties of polyelectrolyte solutions

Here, we describe how we obtain the viscosity of the polyelectrolyte solutions that is used as the viscosity of region (b) (μ_b) and the diffusivity of polyelectrolyte solution used for the effective diffusivity of the charged species. Since the PAA solution is known as a non-Newtonian fluid with yield stress and shear thinning properties[1,2], we measured the zero-shear viscosity of the solutions, which is the viscosity of the fluid when it is effectively at rest, to represent the quiescent state of the region (b) (bulk fluid). To do so, through the rheometer test (ARES-G2, TA instrument Ltd., USA), we measured the shear stress with respect to the shear rate (Supplementary Fig. 11) and obtained the curve of the viscosity to the shear rate (Supplementary Fig. 12). At 0.1 wt% and 0.5 wt%, the PAA solution has a constant viscosity, so we simply choose the constant value of viscosity as the μ_b (Supplementary Fig. 12a, b). In the case of 1.0-2.0 wt% PAA and 0.5 wt% PQ-10 solutions, the viscosity increases as the shear rate decreases, and the viscosity peak appears at the low shear rate regime (Supplementary Fig. 12c-f). This peak represents the zero-shear limit of the viscoplastic fluid[3], so we choose this peak value as the zero-shear viscosity and use it as μ_b (the point that the viscosity decreases without fluctuation, red dotted point in Supplementary Fig. 12c-f). The zero-shear viscosity values in between experimental data (0.25, 0.75, 1.25, 1.75 wt%) were obtained through power regression in two different regimes (< 0.75 wt% and > 0.75 wt%, Supplementary Fig. 13).

Next, we explain how we obtain the diffusivities of the PAA solutions. As the PAA concentration increases in the aqueous solution, the distance between the PAA molecules becomes closer, resulting in an overlap between molecules[4]. The concentration that this molecular "overlap" occurs is called the overlap concentration (c^*), and it determines whether the solution is in dilute regime ($c_p < c^*$, where the c_p is the polyelectrolyte concentration) or in semidilute regime ($c_p > c^*$). The diffusivity of the PAA in the solution varies in these regimes and can be calculated by the following formulas[5]:

Dilute regime: $$D = \frac{8}{3\sqrt{\pi}} \frac{k_B T}{6\pi\mu_0 R_g}, \quad \text{(S. 1)}$$

Semidilute regime: $$D \sim \frac{1}{N_p\sqrt{c_m}}, \quad \text{(S. 2)}$$

where $k_B = 1.38064852 \times 10^{-23}$ m^2 kg s^{-2} K^{-1} is the Boltzmann's constant, $T = 298$ K is

the absolute temperature (room temperature), $\mu_0 = 0.00089$ Pa s is the viscosity of the solvent (water), R_g is the radius of gyration, N_p is the degree of polymerization, and c_m is the monomer concentration (see Supplementary Table 4 for molecular properties of the PAA we used). Equation (S. 1) is the Stokes-Einstein-Zimm formula describing the diffusivity of the polyelectrolyte in the dilute regime with R_g. Equation (S. 2) is the scaling relation of the diffusivity of the polyelectrolyte in the semidilute regime with N_p and c_m[5]. Since we used PAA solutions with various concentrations from 0 to 2 wt% in our experiment, we should first obtain the overlap concentration c^* of PAA to determine the regime of each solution. According to Ying et al.[4], the overlap concentration of PAA is proportional to PAA's molecular weight ($M_w = 104{,}400\ \mathrm{g\ mol^{-1}}$)[6] and R_g^{-3} ($c^* \sim M_w R_g^{-3}$). Here, R_g is estimated as 2.54 nm from the scaling relation $R_g \sim N_p^{0.27}$ in Mintis et al.[7] and $N_p (= 1450)$[6], resulting $c^* \sim a M_w R_g^{-3} \sim 0.85$ wt% (where the coefficient $a = 0.000134$ wt% $\mathrm{nm^3\ mol\ g^{-1}}$ is from Litmanovich et al.[8]).

Now we can separate PAA solutions with two regimes according to $c^* \sim 0.85$ wt% as the dilute regime (0-0.75 wt %) and the semidilute regime (1.0-2.0 wt %).In the dilute regime ($c_p < c^*$) with equation (S. 1), we obtain the constant diffusivity, $D_{PAA} = 1.45 \times 10^{-10}\ \mathrm{m^2 s^{-1}}$, which is independent of c_p. In the semidilute regime ($c_p > c^*$) with equation (S. 2), the monomer concentration is linearly proportional to the PAA concentration ($c_m \sim c_p$), resulting $D_{PAA} \sim N_p^{-1} c_p^{-0.5}$. Therefore, D_{PAA} decreases as c_p increases (see Supplementary Table 5). In the case of the PQ-10 solution, $c^*_{PQ-10} = 3.02 \times 10^{-6}$ wt% ($M_w = 250{,}000\ \mathrm{g\ mol^{-1}}$ in Du et al.[9], $R_g = 223$ nm in Shlar et al.[10], and $N_{p,PQ-10} = 416.67$ (obtained by dividing the median molecular weight into the monomer molecular weight[9])). As c_p of PQ-10 is higher than c^*_{PQ-10}, we used equation (S. 2), resulting $D_{PQ-10} = 1.16 \times 10^{-10}\ \mathrm{m^2\ s^{-1}}$.

In PAA or PQ-10 solutions, the diffusivity of Na^+/Cl^- are presumed not changed in our experiment and scaling analysis. In the experiments with PQ-10, region (a) becomes concentrated with PQ-10 molecules. According to Ariel *et al.*[11], if the size of the substance (e.g., ions) is much smaller than the intermolecular distance of the polymer in the solution, the substance barely interacts with the polymers, so the diffusivity of the substance is the same with that in the solvent without the polymer. In this analogy, PQ-10's radius of gyration (~223 nm) is much larger than the ionic radius of Na^+ / Cl^- (~0.1 nm), and the intermolecular distance is in the order of the radius of gyration even in the high concentration[12]. Therefore, we can assume that the diffusivity of Na^+ and Cl^- in the PQ-10 solution would be constant in our experiment. In the experiments with PAA, since the PAA molecules migrate

toward the anode, the depletion zone (region (a)) is assumed to be a pure water as most of PAA molecules are depleted. Therefore, we adopt the the diffusivity of Na^+ and Cl^- in water, even the PAA's radius of gyration (~2.54 nm) is not much higher than the size of Na^+ /Cl^-, resulting in $D_{Na^+} = 1.33 \times 10^{-9}\ \mathrm{m^2}/s$, $D_{Cl^-} = 2.03 \times 10^{-9}\ \mathrm{m^2}/s$, and $D_{eff} = 2/(1/D_{Na^+} + 1/D_{cl^-}) = 1.6071 \times 10^{-9}\ \mathrm{m^2}/s$, where D_{Na^+} and D_{Cl^-} is the diffusivity of Na^+ and Cl^-, respectively. Viscosities and effective diffusivities of PAA and PQ-10 solutions are summarized in Supplementary Table 1.

Supplementary Note 2: Electroconvective viscous fingering in various polyelectrolytes.

Here we describe the additional condition resulting from experiments with five polyelectrolytes in Supplementary Fig. 5: i) polyacrylic acid (PAA) as a weak anionic polyelectrolyte, ii) polyquaternium-10 (PQ-10) as a strong cationic polyelectrolyte, iii) sodium polystyrene sulfonate (NaPSS) as a strong anionic polyelectrolyte, iv) polyallylamine hydrochloride (PAH) as a weak cationic polyelectrolyte, and v) polyethylene oxide (PEO) as a neutral polymer.

Unlike the finger-like EC appearing in PAA and PQ-10 solutions, the conventional circular EC was observed in 1 wt% PAH and 1 wt% NaPSS solutions, both of which have a marginal viscosity ratio (M=1.9 and 1.66, respectively). We may increase PAH / NaPSS concentrations to achieve a higher viscosity ratio. However, this requires significantly high weight of these polyelectrolytes because their viscosity increase effect is relatively small (Supplementary Fig. 5a). If we increase the concentration to get high bulk viscosity (10 wt% of PAH / NaPSS), the solution's conductance (9.8 / 23.16 mS for PAH / NaPSS 10wt% solution, respectively) is too high to initiate strong ion depletion zone and EC (EC occurs when the concentration of NaCl solution at the membrane surface is sufficiently low for initiating ion depletion zone, and most of EC experiments are conducted under NaCl 0.1 M, where the conductance is 7.3 mS) [13,14]. Consequently, we can find one additional condition to generate EC fingering, i.e., the viscosity increase effect of a polyelectrolyte should be superior to the conductance increase effect according to its concentration.

Supplementary Note 3. The derivation of the finger velocity

Here we describe the governing equations used in our system and detailed derivation of finger velocity (u_0) in the text. To describe our system, we use two governing equations (Momentum equation and Poisson's equation):

$$\rho\left(\frac{\partial \mathbf{U}}{\partial t} + (\mathbf{U}\cdot\boldsymbol{\nabla})\mathbf{U}\right) = -\boldsymbol{\nabla}p + \mu\boldsymbol{\nabla}^2\mathbf{U} - \rho_e\boldsymbol{\nabla}\varphi, \tag{S. 3}$$

$$\rho_e = \boldsymbol{\nabla}\cdot\left(\varepsilon\vec{E}\right) = -\varepsilon\boldsymbol{\nabla}^2\varphi, \tag{S. 4}$$

$$\Rightarrow \rho\left(\frac{\partial \mathbf{U}}{\partial t} + (\mathbf{U}\cdot\boldsymbol{\nabla})\mathbf{U}\right) = -\boldsymbol{\nabla}p + \mu\boldsymbol{\nabla}^2\mathbf{U} + \varepsilon\boldsymbol{\nabla}^2\varphi\boldsymbol{\nabla}\varphi, \tag{S. 5}$$

where ρ is a density, $\mathbf{U}$ is velocity vector, p is a pressure, μ is a viscosity, ρ_e is a charge density, φ is an electrical potential, ε is a permittivity and $\vec{E}$ is an electric field. Without external pressure and inertia term in (S. 8), region (a) in the text (the depletion zone) can be described with the Stokes equation, which is

$$0 = \mu_a\boldsymbol{\nabla}^2\mathbf{U}_a + \varepsilon_a\boldsymbol{\nabla}^2\varphi_a\boldsymbol{\nabla}\varphi_a. \tag{S. 6}$$

The subscript a indicates that the property is of the region (a) (so is subscript b in the region (b) below). Scaling with $U_a \sim u_{EC}$, $\varphi_a \sim \varphi_{EC}$, and $x \sim d_{EC}$, we can get

$$u_{EC} \sim \frac{\varepsilon_a\varphi_{EC}^2}{\mu_a d_{EC}}, \tag{S. 7}$$

where u_{EC} is the velocity of EC in the region (a), φ_{EC} is the electrical potential across the depletion zone, and d_{EC} is the size of EC. If we scale the finger velocity ($u_0 = \partial d_{EC}/\partial t$) as the u_{EC}, we can get the u_0 value by the following mathematical process:

$$u_{EC} \sim \frac{\varepsilon_a\varphi_{EC}^2}{\mu_a d_{EC}} \sim \frac{\partial d_{EC}}{\partial t} = u_0, \tag{S. 8}$$

$$\int d_{EC}\,\partial d_{EC} \sim \int \frac{\varepsilon_a\varphi_{EC}^2}{\mu_a}\partial t \;\Rightarrow\; d_{EC} \sim \sqrt{\frac{\varepsilon_a\varphi_{EC}^2}{\mu_a}t}, \tag{S. 9}$$

$$u_0 = \frac{\partial d_{EC}}{\partial t} \sim \sqrt{\frac{\varepsilon_a\varphi_{EC}^2}{\mu_a t}}. \tag{S. 10}$$

Supplementary Note 4. pH/PAA concentration profiling

The pH change (1.5-6) and the PAA concentration (0-2 wt%) are represented by the gray value (Supplementary Fig. 4a). The correlation between pH and gray value is illustrated in the methods section in the manuscript. There are three regions according to the pH/PAA concentration profiles: i) diffusive ion enrichment zone on the top CEM with a linear increase of PAA concentration, ii) bulk region where the initial pH/concentration maintained (i.e., region (b) in Fig.1c), and iii) ion depletion zone on the bottom CEM with EC and corresponding flat pH/concentration profiles (i.e., region (a) in Fig.1c). During 8 sec operations at the applied voltage of 30 V in 0.5 wt% PAA solution, the diffusive enrichment zone expands only about 0.2 mm (which is well matched with the theoretical diffusion length, 0.1 mm $\sim\sqrt{4D_{PAA}t}$, see Supplementary Fig. 4b): whereas EC fingering grows much faster up to ~1 mm (Supplementary Fig. 4c-d). Interestingly, we can observe the EC-induced current hotspot between the fingers where the influx of the vortices occurs (white arrows in Supplementary Fig. 4a)[15]. At this point, not only the depletion zone is suppressed, but also PAA is concentrated (Supplementary Fig. 4d). Accordingly, as the viscosity between the fingers increases, downward flows between the fingers are considerably suppressed whereas there are upward flows in the fingers. Such phenomenon is strengthened when EC are densely packed (e.g., 0.5 wt%, 30 V in Supplementary Video 1).

As described above, there are spatiotemporal variations of pH /PAA concentration in the ion enrichment /depletion zones during the developing EC fingers. However, these variations, except for change at the bulk-depletion region, are negligible for the following three reasons. First, the bulk region completely separates the ion enrichment and depletion zones until EC touches the enrichment zone after a considerable time (e.g., > 14 sec at the applied voltage of 30 V in 0.5 wt% PAA solution); at this merging moment, EC already determine its shape. Consequently, we can assume that the region (a) and (b) have constant viscosities. Also, while the viscosity between EC vortices increases as the PAA concentration increases, this is just one of the consequences of EC after it emerged. EC shape (circular or straight or ramified fingers) is still determined by the viscosity gradient of the ion depletion zone and the bulk region (i.e., viscosity ratio M). In addition, we estimated shear rates from the particle tracking images, and confirmed that it is mostly below 1 s^{-1} (Supplementary Fig. 2). Referring to Supplementary Fig. 12, the shear-thinning effect is not significant at below 1 s^{-1} shear rate, so we also can neglect the viscosity change by shear thinning of polyelectrolytes induced by EC vortices. Lastly, the pH variation in whole regions is between 1.5 to 4.5 (Supplementary Fig. 4). Therefore, we can expect the PAA molecules to be in a globular form and the radius of gyration is nearly constant everywhere[16].

Supplementary Note 5. The derivation of the Deborah number

To scale Deborah number (De=$t_{relax}/t_{process}$, where t_{relax} is the relaxation time, and $t_{process}$ is the processing time), the relaxation time represents as $t_{relax} = \mu_b/G$[17], where the shear modulus of PAA solutions follows the scaling relation as $G \sim c_m^{7/12} c_s^{1/4} k_B T/N_p$[18,19] ($c_m$ is the monomer concentration (number density), c_s is the salt concentration, k_B is the Boltzmann's constant, T is the temperature, and N_p is the degree of polymerization). The viscosity μ_b is represented to $0.0272 c_p^{7.0918}$ in Supplementary Fig. 13 (c_p is the concentration of the PAA solution). After rearranging, we can present $t_{relax} \sim \mu_b^{0.917}$ with constant c_m and N_p in our experiments. The processing time is the exposure time that the viscosity gradient boundary is exposed /affected to EC vortices, resulting $t_{process} \sim d_{EC}/u_{EC} \sim \mu_a d_{EC}^2/\varepsilon_1 \varphi_{EC}^2$ ($\because$ $u_{EC} \sim \varepsilon_a \varphi_{EC}^2/\mu_a d_{EC}$ as we used the derivation of the equation (1)). By substituting two scaling results, i.e., $t_{relax} \sim \mu_b^{0.917}$ and $t_{process} \sim \mu_a d_{EC}^2/\varepsilon_1 \varphi_{EC}^2$, we obtain the scaling result as $De \sim M^{0.917} \varphi_{EC}^2/d_{EC}^2$ (the equation (5)).

Supplementary Figures

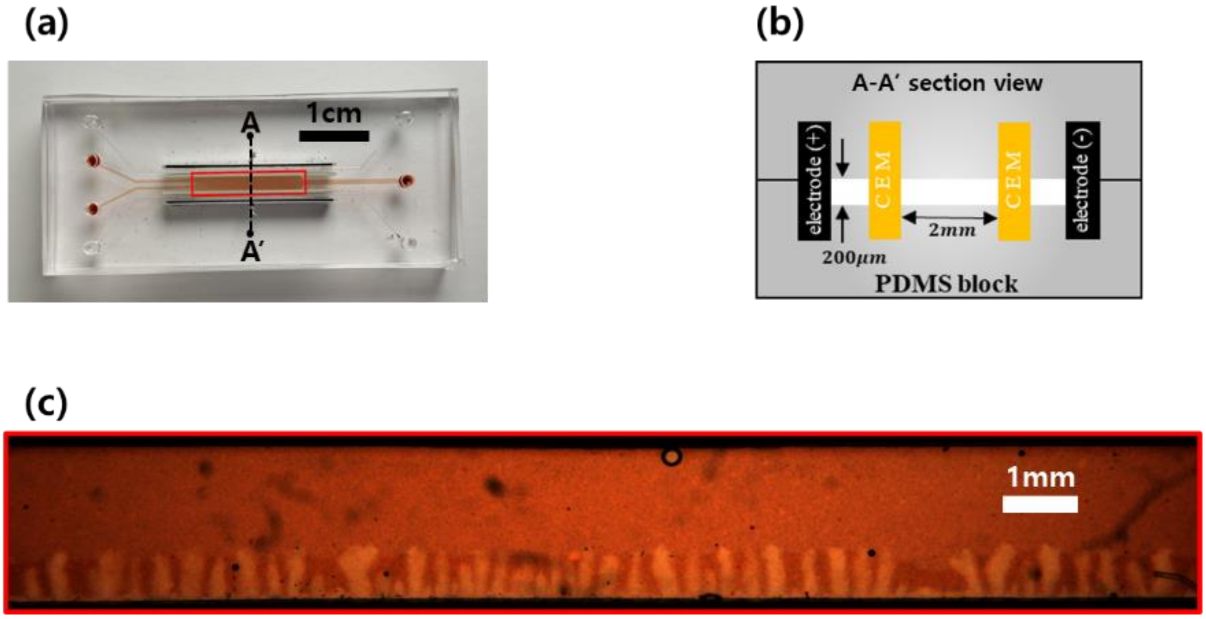

Supplementary Figure 1. The visualization device. a, Picture of the visualization device. **b,** Schematic of the device (cross-sectional view). The main channel for the visualization is between the cation exchange membranes (CEMs), and two side channels are between the CEM and the electrode. **c,** Example of the experimental image with applied voltage of 30 V in 1 wt% PAA solution (red rectangular region of **a**).

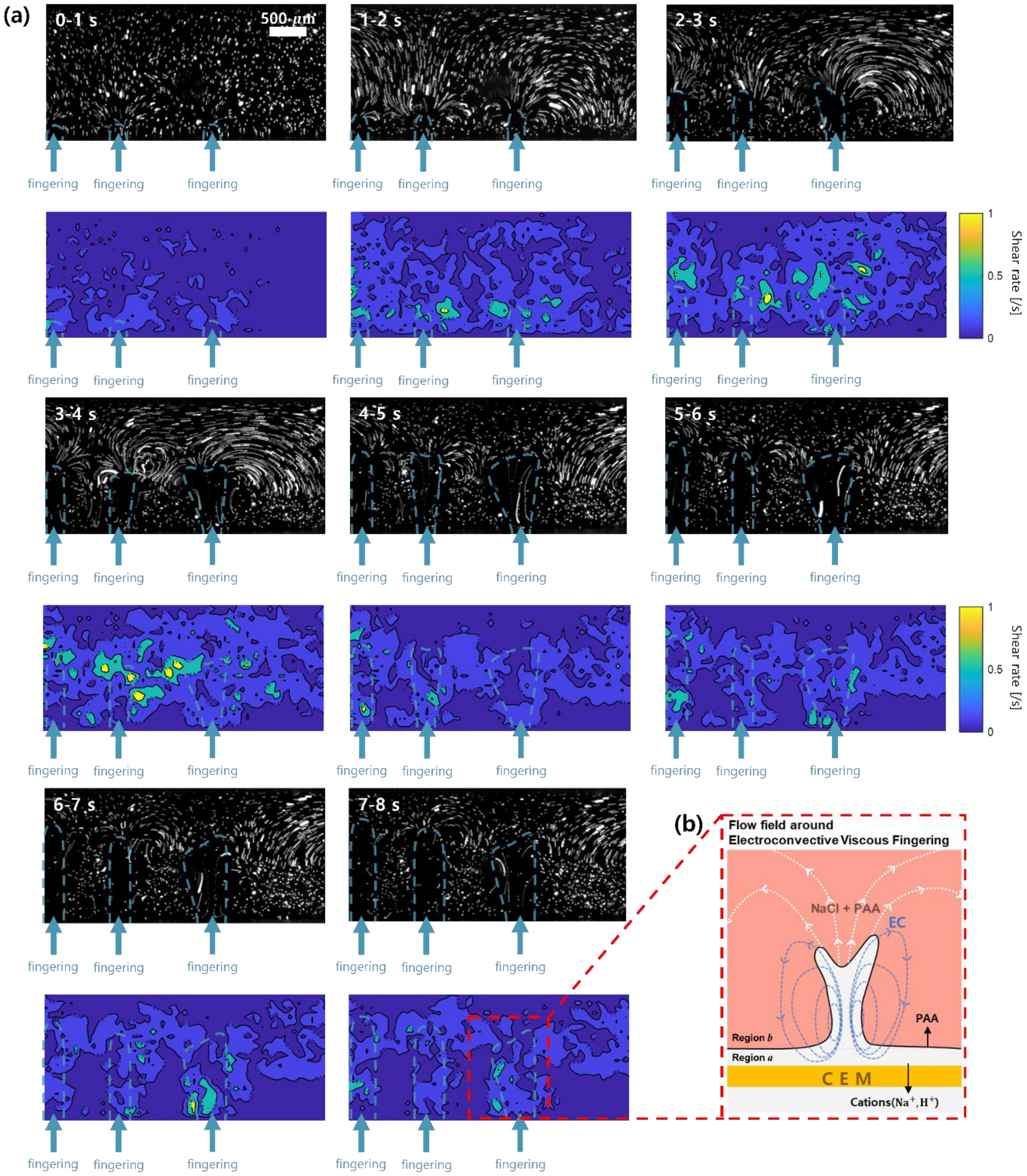

Supplementary Figure 2. Velocity profile of the electroconvective viscous fingering at 30V in 1.0wt% PAA solution. a, Stacked images of electroconvective viscous fingering with 10 μm fluorescent particles (FluoSpheres™ polystyrene, Invitrogen, CA). The CEM is located at the bottom of the images. **b,** Schematic image of the flow field around the electroconvective viscous fingering. As EC in region (a) (white region) penetrates into region (b) (red region), electroconvective viscous fingering occurs and the fluid flows across the fingers in a vortex shape. The images were taken at a speed of 20 frames per second and stacked with 20 frames (1 s). Shear rate images are represented below the stacked images, which shows the shear rate remains below a nearly 1 s^{-1}. This demonstrates the shear-thinning effect is trivial to the growth of finger. We can observe the clear three streams of the fingers and vortex fields around them.

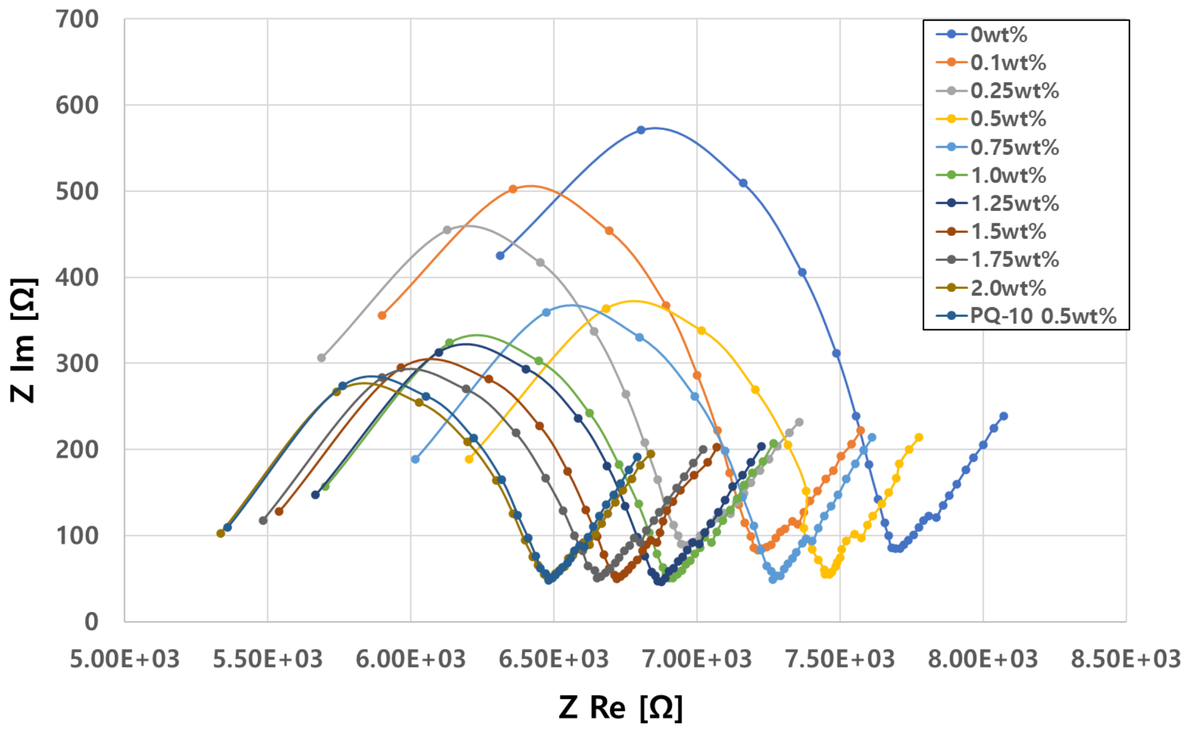

Supplementary Figure 3. Nyquist plots of polyelectrolyte solutions. We measured electrochemical impedance spectroscopy in the frequency range of 10^6-10^3 Hz performed with PalmSens 4 (PalmSens BV, Houten, the Netherlands). The x and y-axis indicate the real part and imaginary part of the complex resistance.

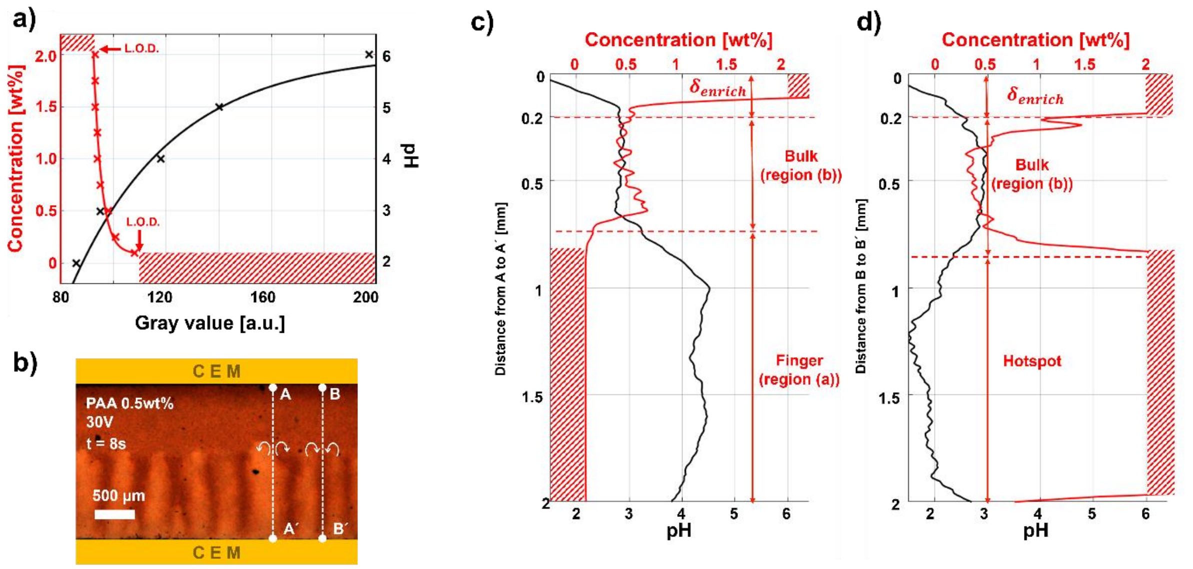

Supplementary Figure 4. pH and PAA concentration profiles with electroconvective viscous fingering. a, Correlations between pH, PAA concentration and the gray value in microscopic images. The red line represents the correlation of the gray value with PAA concentration, and the black line represents the correlation of the gray value with pH. The X marks represent the experimental data, and the solid lines represent the best-fit lines. Here, we can identify the upper / lower limit of detections of PAA concentration as > 2 wt% and < 0.1 wt%. **b**, The experimental image captured when the finger sufficiently develops (after 8 s since the voltage applied). **c-d**, pH and PAA concentration variations across the finger (A-A' in (b)) and between the finger (i.e., current hotspot, B-B' in (b)), respectively.

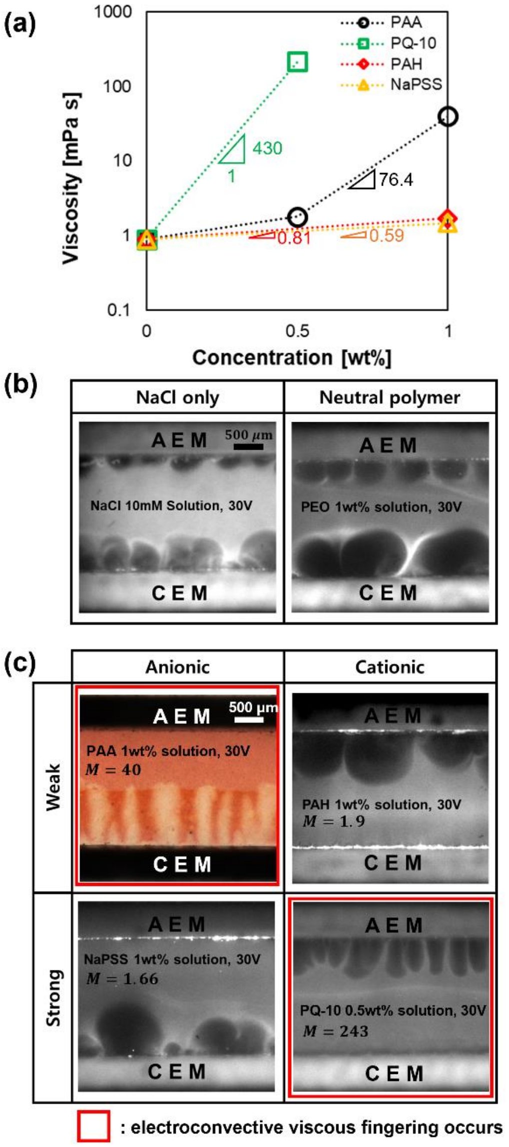

Supplementary Figure 5. EC in various types of polyelectrolyte/polymer on AEM and CEM. a, Viscosities of the four polyelectrolyte solutions (neutral: polyethylene oxide (PEO), weak anionic: polyacrylic acid (PAA), strong anionic: sodium polystyrene sulfonate (NaPSS), weak cationic: polyallylamine hydrochloride (PAH), and strong cationic: Polyquaternium-10 (PQ-10)). The viscosities of the solutions were measured as 1.48, 1.7, 40, and 216 mPa s for 1wt% NaPSS, PAH, PAA, and 0.5wt% PQ-10 solution, respectively. The viscosity of PEO 1wt% solution is 2.2 mPa s, which is not displayed in graph. **b,** Microscopic images of EC in 10 mM NaCl solution and in the neutral polymer solution (1 wt% PEO). The circular EC vortices appear on both AEM and CEM. **c,** Microscopic images with anionic/cationic weak/strong polyelectrolytes. 1 wt% of PAA, NaPSS, PAH, and 0.5 wt% of PA-10 solutions were used. The polyelectrolytes suppress the EC on the side where they are concentrated under the electric field, while the neutral polymer does not at moderate concentration (1wt%). The electroconvective viscous fingering emerges in PAA and PQ-10 solutions ($M = 40$ and 243, respectively), where the viscosity increases significantly with their concentration. However, the EC in PAH and NaPSS solutions ($M = 1.9$ and 1.66, respectively) exhibits a circular EC shape.

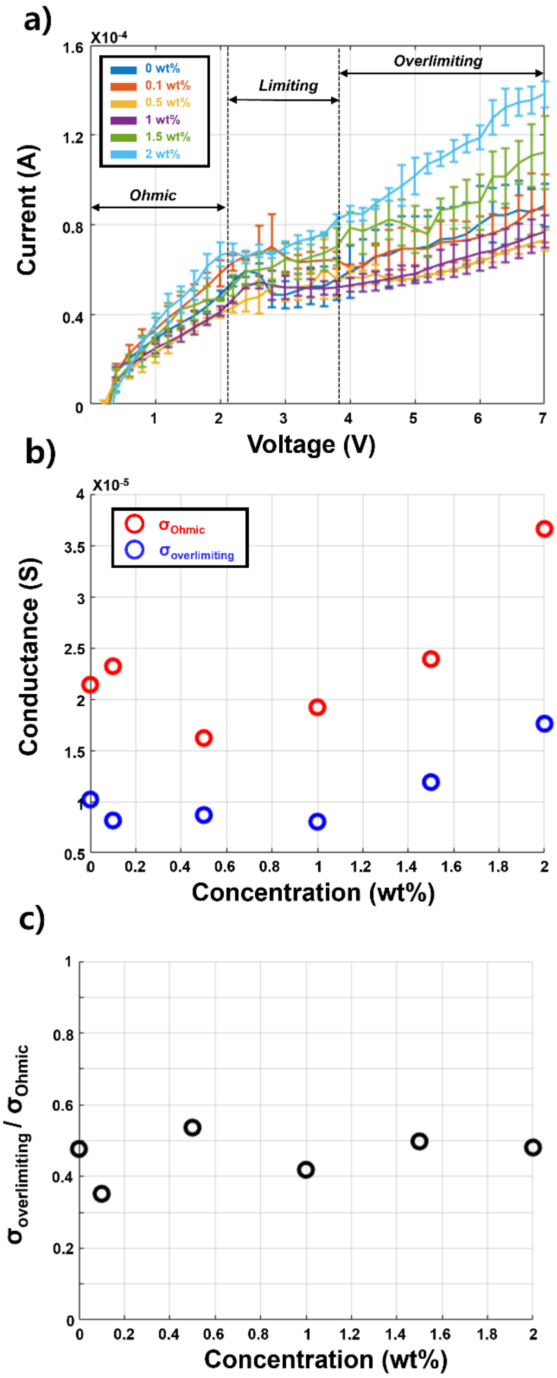

Supplementary Figure 6. The current-voltage responses and conductances in various PAA concentrations (0-2.0 wt%) a, Current-voltage curves according to various PAA concentrations (0-2.0 wt%). Ohmic, limiting, and overlimiting regimes are clearly observed in all cases. Each case is conducted three times, and the error bar represents to the standard deviation. **b**, Ohmic conductance (σ_{Ohmic}) and overlimiting conductance ($\sigma_{overlimitig}$) and **c,** the ratio of them according to PAA concentrations. σ_{Ohmic} is the slope of current-voltage curve in the Ohmic regime, and $\sigma_{Overlimiting}$ is that in the overlimiting regime. By the release of PAA's counter ion (i.e., H^+), two conductance start to increase at > PAA 1.0 wt%.

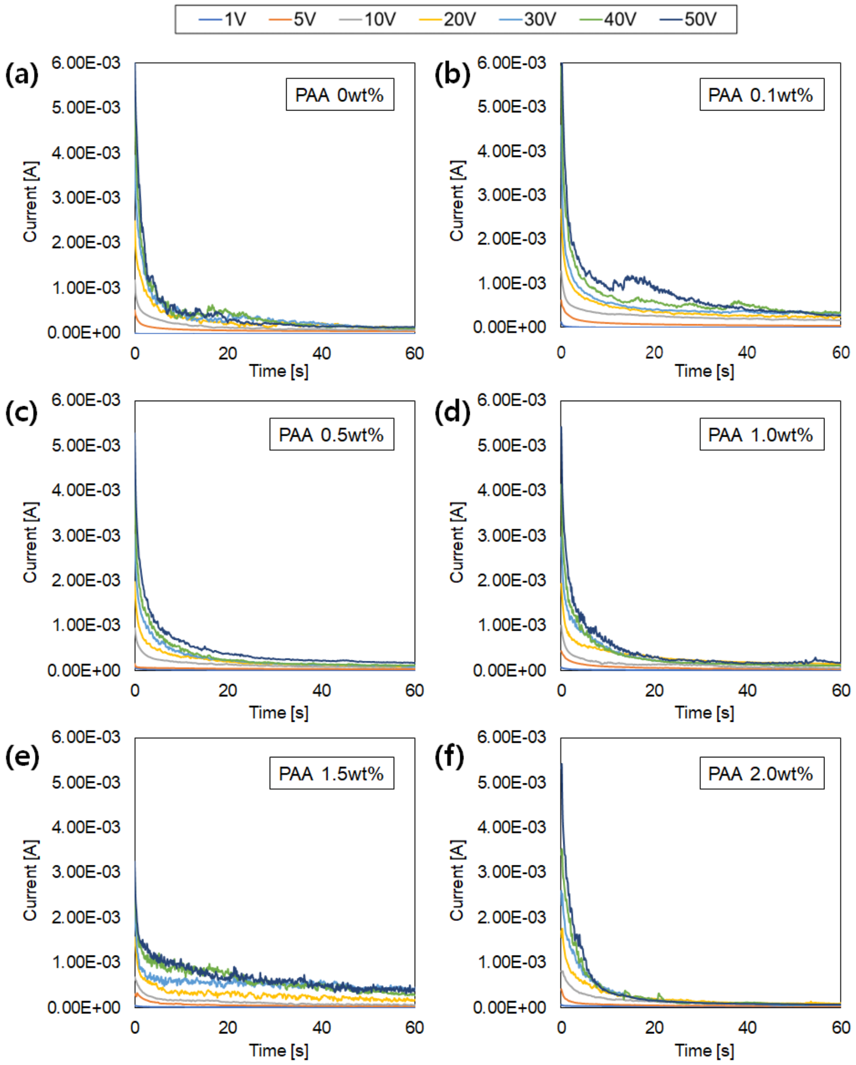

Supplementary Figure 7. a-f, Current-time curves according to various PAA concentrations (0-2.0 wt%) and various applied voltages (1-50 V). Current response was measured for 60 s.

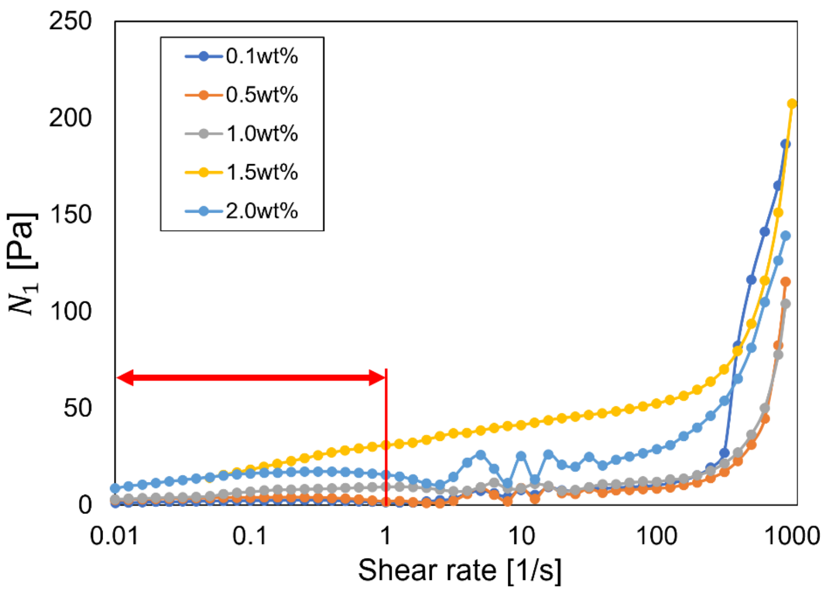

Supplementary Figure 8. The first normal stress difference (N_1) vs. shear rate curve of PAA solutions. The rheometer test was conducted with ARES-G2 Rheometer (TA instruments, New Castle, DE) in the shear rate range of 0.01-900 1/s. The averaged N_1 used in Fig. 4 in the manuscript is averaged in the shear rate range of < 1 s^{-1}, which is depicted as a red arrow.

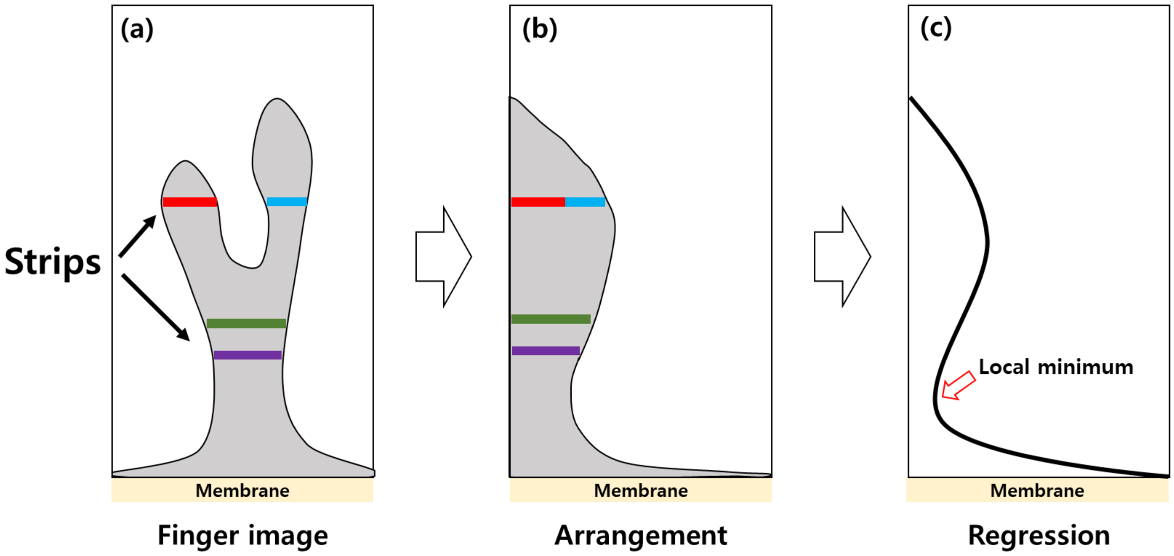

Supplementary Figure 9. Method for measuring the critical EC length. There are three steps for measuring the critical EC length ($d_{EC,critical}$) from the experimental images. **a**, First, we prepare each finger image of experiments. **b**, Second, we rearrange the finger image by accumulating the width of the finger on the left side of the box (each horizontal strip in a and **b** is identical). **c**, Lastly, we regress the finger's boundary by 3rd degree polynomial model. Here, the critical EC length is determined by the distance from the membrane to the local minimum of the finger. Since each experimental case has more than one finger, the median value is obtained for $d_{EC,critical}$ in Fig. 5b.

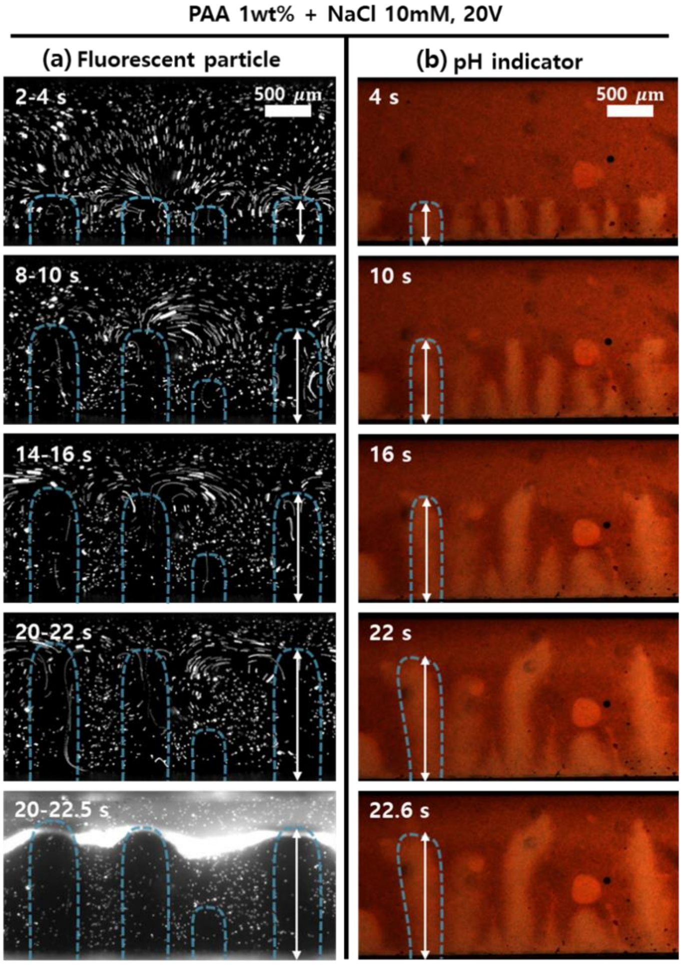

Supplementary Figure 10. Verification of the visualization reliability of EC. a, Stacked images of electroconvective viscous fingering at the applied voltage of 20 V in 1.0 wt% PAA solution with 10 μm fluorescent particles. The CEM is located on the bottom of the images. The images were taken at a speed of 20 frames per second and stacked with 40 frames (2 s). The anionic fluorescent dye (1.2 uM Alexa Fluor 488) is also dissolved in the solution and visualized at the specific time (t=22.5s). The experiment was conducted in the main channel of the cell (Supplementary Fig. 1c). **b,** Visualization images with pH indicator (Hydrion One Drop, Micro Essential Laboratory Inc., USA) taken in the same condition as **a**. In **a**, the fluorescent particles can follow the fluid flows (bright dots), but the structure of the finger is not clearly visualized, since the negatively charged fluorescent dyes are pushed away from the membrane and not coming back as shown in **a** (t=22.5s). In **b**, however, finger structures can be visualized with pH indicator in the red index. This is because the pH indicator can visualize the hydrogen ion, which is the counter ion of the PAA, so it can also track the PAA in the cell. As a result, the pH indicator visualizes the development of two regions; the bulk electrolyte as the relatively red region (region (b) in Fig. 1c and the ion depletion zone as the relatively white region (region (a) in Fig. 1c).

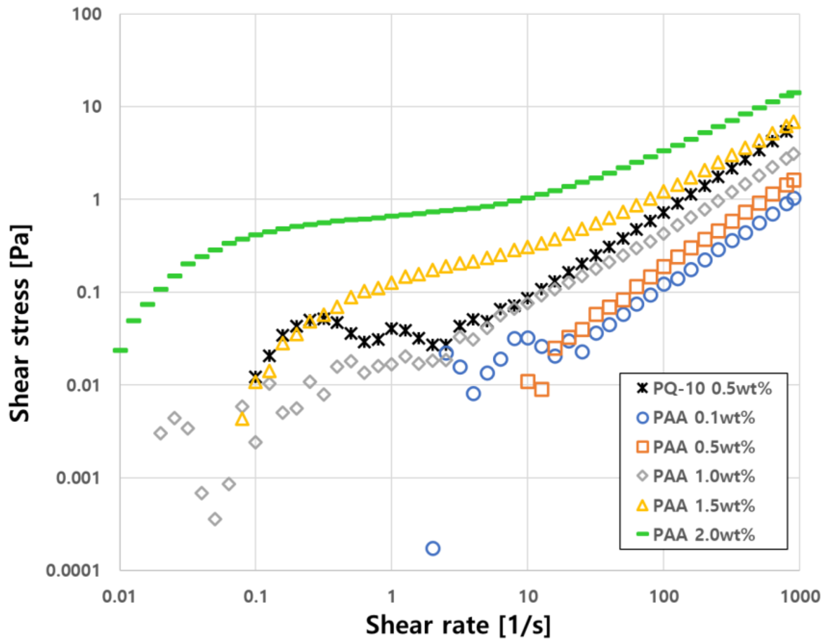

Supplementary Figure 11. Shear stress vs. shear rate curve of polyelectrolyte solutions. The rheometer test was conducted with ARES-G2 Rheometer (TA instruments, New Castle, DE) in the shear rate range of 0.01-900 1/s.

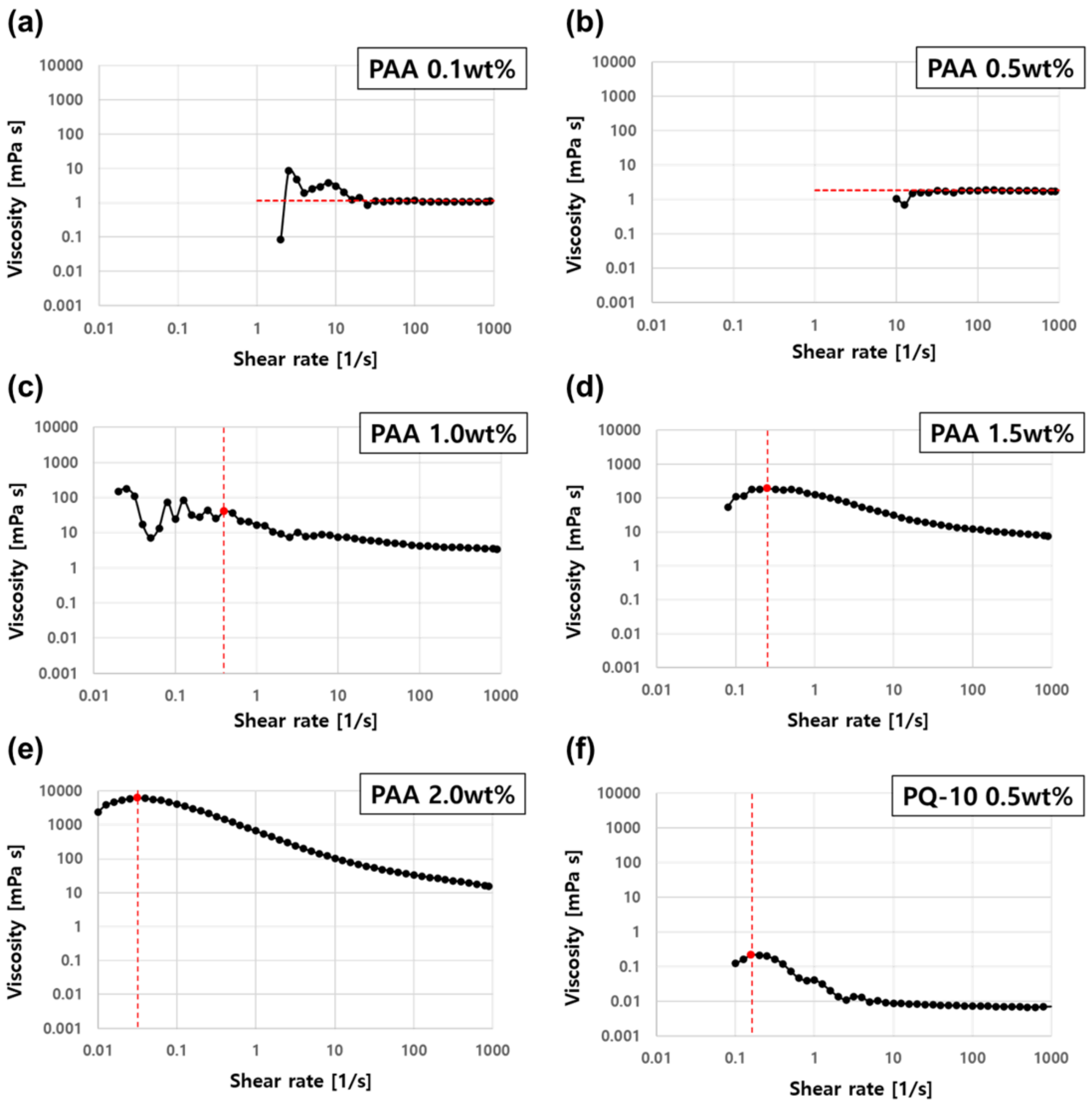

Supplementary Figure 12. Viscosity vs. shear rate curve of polyelectrolyte solutions. a-b, At 0.1 wt% and 0.5 wt% PAA solutions, we can see the plateau of the curve at the shear rate > 10 s^{-1}. **c-f**, At 1-2 wt% PAA and 0.5 wt% PQ-10 solutions, the viscosity peaks are identified at the shear rate < 1 s^{-1}, which is one of the representative properties of a yield stress fluid. The data of these graphs are obtained from the rheometer tests in Supplementary Figure 11.

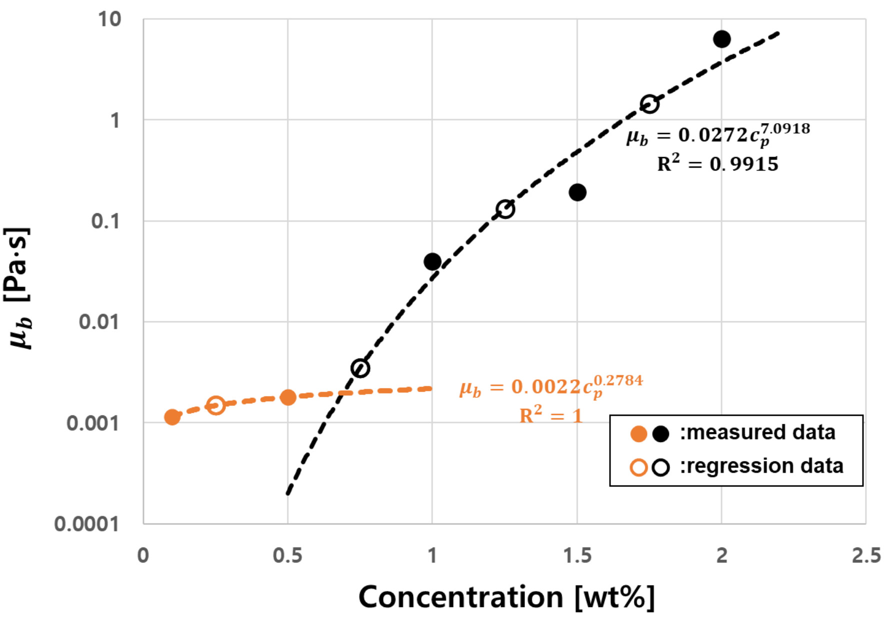

Supplementary Figure 13. Zero-shear viscosities of PAA solutions. The zero-shear viscosities are obtained via the rheometer tests for 0.1, 0.5, 1, 1.5, and 2 wt% PAA solutions (Supplementary Figure 11-12). To obtain the zero-shear viscosity at 0.25, 0.75, 1.25, and 1.75 wt% PAA solution, the regression curves are used for two separate regimes (Newtonian fluid < 0.5 wt%, yield stress fluid: > 0.5 wt%). The zero-shear viscosities from those regression curves are addressed in Supplementary Table 1.

Supplementary Tables

Supplementary Table 1. Viscosity and effective diffusivity of 10 mM NaCl solutions with 0-2.0 wt % of PAA and 0.5 wt% of PQ-10. Viscosity was obtained with the viscosity-shear rate curve obtained from the rheometer tests (Supplementary Fig. 11-13). The asterisk marked values (*) were obtained through regression. The diffusivity of PAA and PQ-10 was obtained by the theoretical formula and scaling relation[5], and calculated the effective diffusivity with that of Na^+ and Cl^- at water (Supplementary Note 1).

wt% of PAA	μ_b $[Pa\ s]$	D_{eff} $[m^2s^{-1}]$
0	0.00089	1.61E-09
0.1	0.00115	
0.25	*0.001496	
0.5	0.0018	
0.75	*0.003536	
1.0	0.03981	
1.25	*0.1324	
1.5	0.1934	
1.75	*1.439	
2.0	6.344	
PQ-10 0.5wt%	0.2162	2.03E-09

Supplementary Table 2. Viscoplastic coefficients of 1.0-2.0 wt% PAA solutions obtained from the Herschel-Bulkley model. Raw data for regression is obtained from the result in Supplementary Fig. 11. τ is the shear stress, τ_y is the yield stress, K is the consistency index, $\dot{\gamma}$ is the shear rate, and n is the flow index.

Regression to Herschel-Bulkley model $\tau = \tau_y + K \cdot \dot{\gamma}^n$				
**wt% of PAA**	**Coefficients (with 95% confidence bounds)**			**Goodness of fit**
	τ_y [Pa]	K [Pa s^n]	**n**	R^2
**1.0**	0.01173 (0.008017, 0.01543)	0.00647 (0.006142, 0.006798)	0.9062 (0.8985, 0.9139)	0.9998
**1.5**	0.08363 (0.06657, 0.1007)	0.02798 (0.02572, 0.03024)	0.8075 (0.7952, 0.8198)	0.9995
**2.0**	0.3722 (0.3179, 0.4265)	0.1271 (0.1129, 0.1412)	0.689 (0.672, 0.7061)	0.9985

Supplementary Table 3. Lists of symbols and dimensionless parameters

Symbols	Description	Value	Units
N	The number of fingers	-	-
c_0	Bulk ion concentration (of NaCl)	10E-6	mol L^{-1}
c_p	Polyelectrolyte concentration (of PAA or PQ-10)	0 – 2.0	wt%
h	Channel height	2E-06	m
w	EC vortex width	-	m
L	EC vortex length	-	m
d_{EC}	Size of the electroconvection	-	m
$d_{EC,critical}$	Critical size of electroconvection	-	m
A_1	Area of the finger	-	m^2
A_2	Area of the bounded rectangle of the finger	-	m^2
ρ	Fluid density	-	kg m^{-3}
μ	Dynamic viscosity	-	kg m^{-1} s^{-1}
ν	Kinematic viscosity ($= \mu/\rho$)	-	m^2 s^{-1}
$\boldsymbol{U}$	Velocity vector	-	m s^{-1}
u_{EC}	Velocity of electroconvection ($= \varepsilon_a \varphi_{EC}^2/(\mu_a D_{eff})$)	-	m s^{-1}
u_0	EC interface development speed ($= \sqrt{\varepsilon_a \varphi_{EC}^2/(\mu_a t)}$)	-	m s^{-1}
p	Pressure	-	kg m^{-1} s^{-2}
ρ_e	Charge density	-	C m^{-3}
ε_a	Electric permittivity in region (a) (for the PAA solution)	6.95E-10	C^2 s^2 kg^{-1} m^{-3}
ε_{PQ-10}	Electric permittivity (for the PQ-10 solution)	3.54E-10	C^2 s^2 kg^{-1} m^{-3}
φ	Electrical potential	-	kg m^2 s^{-2} C^{-1}
φ_{EC}	Electrical potential across the depletion zone ($= I(R - R_{ohmic})$)	-	kg m^2 s^{-2} C^{-1}
I	Current	-	C s^{-1}
R	Total resistance of the cell	-	Ω
R_{ohmic}	Ohmic resistance of the cell	-	Ω
D_{Na^+}	Diffusion coefficient of Na^+	1.33E-09[20]	m^2 s^{-1}
D_{Cl^-}	Diffusion coefficient of Cl^-	2.03E-09[20]	m^2 s^{-1}
D_{PAA}	Diffusion coefficient of PAA	-	m^2 s^{-1}
D_{eff}	Effective diffusivity of charged species ($= 3/(1/D_{Na^+} + 1/D_{Cl^-} + 1/D_{PAA})$)	-	m^2 s^{-1}
t_{relax}	Relaxation time	-	s
$t_{process}$	Processing time	-	s

Dimensionless parameters	Description	Formula
Ra_E	Electric Rayleigh number	$\varepsilon_a \varphi_{EC}/(\mu_a D_{eff})$
M	Viscosity ratio	μ_b/μ_a
M_ρ	Density ratio	ρ_b/ρ_a
Sc	Schmidt number	ν_a/D_{eff}
S_1	Aspect ratio of the finger	L/w
S_2	Area fraction of the finger to the bounded rectangle	A_1/A_2
De	Deborah number	$t_{relax}/t_{process}$
$\tilde{\boldsymbol{U}}$	Dimensionless velocity vector	$\boldsymbol{U}/u_0$
$\tilde{p}$	Dimensionless pressure	$p/(\mu_b u_0/h)$

*In all symbols, the subscript a and b indicates that the property is of the region (a) and (b).

*In all symbols, the tilde denotes dimensionless variables.

Supplementary Table 4. Molecular properties of the polyacrylic acid (PAA). M_w is the molecular weight of the PAA[6], N_p is the degree of polymerization[6], $M_{monomer}$ is the molecular weight of the PAA monomer , c^* is the overlap concentration[8], and R_g is the radius of gyration[7]

M_w[g mol^{-1}]	N_p	$M_{monomer}$[mol^{-1}]	c^*[wt%]	R_g[nm]
104,400[6]	1450[6]	72 ($C_3H_4O_2$)	0.84786	2.5432

Supplementary Table 5. Diffusivities of PAA, PQ-10, Na^+ and Cl^-. The diffusivities of PAA and PQ-10 are obtained from the theoretical formula and scaling relations (see Supplementary Note 1).

wt%	0.1~0.75	1.0	1.25	1.5	1.75	2.0	PQ-10 0.5wt%	Na^+	Cl^-
Diffusivity [× 10^{-10}m^{2}s^{-1}]	1.45	1.34	1.19	1.09	1.01	0.945	1.16	13.3	16.1

References of Supplementary Information

1 Curran, S. J., Hayes, R. E., Afacan, A., Williams, M. C. & Tanguy, P. A. Properties of carbopol solutions as models for yield-stress fluids. *J Food Sci* **67**, 176-180, doi:DOI 10.1111/j.1365-2621.2002.tb11379.x (2002).

2 Wang, Y., Pethrick, R. A., Hudson, N. E. & Schaschke, C. J. Rheology of poly (acrylic acid): A model study. *Industrial & engineering chemistry research* **51**, 16196-16208 (2012).

3 Souza Mendes, P. R. & Dutra, E. S. Viscosity function for yield-stress liquids. *Applied Rheology* **14**, 296-302 (2004).

4 Ying, Q. C. & Chu, B. Overlap Concentration of Macromolecules in Solution. *Macromolecules* **20**, 362-366, doi:DOI 10.1021/ma00168a023 (1987).

5 Muthukumar, M. Dynamics of polyelectrolyte solutions. *J Chem Phys* **107**, 2619-2635, doi:Doi 10.1063/1.474573 (1997).

6 Carnali, J. O. & Naser, M. S. The Use of Dilute-Solution Viscometry to Characterize the Network Properties of Carbopol Microgels. *Colloid Polym Sci* **270**, 183-193, doi:Doi 10.1007/Bf00652185 (1992).

7 Mintis, D. G. & Mavrantzas, V. G. Effect of pH and Molecular Length on the Structure and Dynamics of Short Poly(acrylic acid) in Dilute Solution: Detailed Molecular Dynamics Study. *J Phys Chem B* **123**, 4204-4219, doi:10.1021/acs.jpcb.9b01696 (2019).

8 Litmanovich, E. A., Zakharchenko, S. O. & Stoichev, G. V. Influence of chain charge and complexation on the overlap and entanglements formation in poly(acrylic acid) salt-containing aqueous solutions. *J Phys Chem B* **111**, 8567-8571, doi:10.1021/jp070070t (2007).

9 Du, C. S., Ho, I. H., Huang, Y. J. & Lee, R. H. Quaternary ammonium halide-containing cellulose derivatives for defect passivation in MAPbI(3)-based perovskite solar cells. *Sustain Energ Fuels* **6**, 3349-3362, doi:10.1039/d2se00516f (2022).

10 Shlar, I. *et al.* High-Throughput Screening of Nanoparticle-Stabilizing Ligands: Application to Preparing Antimicrobial Curcumin Nanoparticles by Antisolvent Precipitation. *Nano-Micro Lett* **7**, 68-79, doi:10.1007/s40820-014-0020-6 (2015).

11 Michelman-Ribeiro, A., Horkay, F., Nossal, R. & Boukari, H. Probe diffusion in aqueous poly (vinyl alcohol) solutions studied by fluorescence correlation spectroscopy. *Biomacromolecules* **8**, 1595-1600 (2007).

12 Dobrynin, A. V., Jacobs, M. & Sayko, R. Scaling of polymer solutions as a quantitative tool. *Macromolecules* **54**, 2288-2295 (2021).

13 Nikonenko, V. V. *et al.* Intensive current transfer in membrane systems: Modelling, mechanisms and application in electrodialysis. *Advances in colloid and interface science* **160**, 101-123 (2010).

14 Zabolotsky, V. *et al.* Coupled transport phenomena in overlimiting current electrodialysis. *Separation and purification technology* **14**, 255-267 (1998).

15 Stockmeier, F. *et al.* Direct 3D observation and unraveling of electroconvection phenomena during concentration polarization at ion-exchange membranes. *J Membrane Sci* **640**, 119846 (2021).

16 Laguecir, A. *et al.* Size and pH effect on electrical and conformational behavior of poly (acrylic acid): Simulation and experiment. *European polymer journal* **42**, 1135-1144 (2006).

17 Kamani, K., Donley, G. J. & Rogers, S. A. Unification of the rheological physics of yield stress fluids. *Physical review letters* **126**, 218002 (2021).

18 Jaworski, Z., Spychaj, T., Story, A. & Story, G. Carbomer microgels as model yield-stress fluids. *Reviews in Chemical Engineering* (2021).

19 Rubinstein, M., Colby, R. H., Dobrynin, A. V. & Joanny, J.-F. Elastic modulus and equilibrium swelling of polyelectrolyte gels. *Macromolecules* **29**, 398-406 (1996).

20 Mills, R. & Lobo, V. M. *Self-diffusion in electrolyte solutions: a critical examination of data compiled from the literature*. (Elsevier, 2013).
