# Supplementary material for: Electroconvective viscous fingering in a single polyelectrolyte fluid on a charge selective surface
Source: Nat Commun. 2023 Nov 17;14:7455. doi: 10.1038/s41467-023-43082-9 (PMC10656491; doi:10.1038/s41467-023-43082-9)
Supplement: Supplementary file 3 — Description of Additional Supplementary Files [file 41467_2023_43082_MOESM3_ESM.pdf]

## Description of Additional Supplementary Files

**File Name: Supplementary Movie 1**
**Description:** Representative experimental video of electroconvective viscous fingering in various concentrations of PAA under 30 V.

**File Name: Supplementary Movie 2**

**Description:** The generation process of the ramified electroconvective viscous fingering.
